# Supplementary material for: Dietary Protein Intake and Transition between Frailty States in Octogenarians Living in New Zealand
Source: Nutrients. 2021 Aug 19;13(8):2843. doi: 10.3390/nu13082843 (PMC8401514; doi:10.3390/nu13082843)
Supplement: Supplementary file 1 [file nutrients-13-02843-s001.zip › nutrients-1305094-supplementary.pdf]

nutrients

MDPI

Supplementary tables and figures

Table S1. Characteristics of those with and without the variables needed to assign a frailty state at wave 2.

	without Frailty state	with Frailty state	*p*
N	139	542	
Women, n (%)	76 (54.7)	299 (55.2)	0.994
Age (years)	84.7 (2.3)	85.4 (1.9)	<0.001
Māori % (n)	92 (66.2)	186 (34.3)	<0.001
Education, n (%)			0.509
Primary or no schooling	36 (26.9)	115 (21.4)	
Secondary school, no qualification	49 (36.6)	193 (35.9)	
Secondary school, qualification	27 (20.1)	109 (20.3)	
Trade, occupational	10 (7.5)	50 (9.3)	
Tertiary qualification	12 (9.0)	70 (13.0)	
Rest home/ private hospital, n (%)	0 (0.0)	55 (10.1)	0.001
Disease burden	2.9 (2.1)	3.1 (2.0)	0.573
Body weight (kg)	75.0 (14.1)	71.3 (13.8)	0.089
BMI (kg/m^2)	29.1 (5.6)	27.4 (4.9)	0.028
Energy intake (MJ/d)	6.5 (2.6)	7.1 (2.4)	0.054
Protein intake (g/d)	65.2 (28.7)	66.6 (23.7)	0.659
Protein intake (g/ kg BW/d)	0.92 (0.41)	0.96 (0.34)	0.479
≥ 0.8 g/ kg BW/d, n (%)	24 (54.5)	316 (65.3)	0.207
≥ 1.0 g/ kg BW/d, n (%)	16 (36.4)	191 (39.5)	0.809
Carbohydrate intake (g/d)	166 (73)	186 (59)	0.009
Fat intake (g/d)	69.0 (33.4)	71.4 (33.4)	0.572

Entries are means ± SD unless mentioned. Ethnicity refers to Māori and non-Māori. Co-morbidity is the sum of 19 chronic diseases and was measured at wave 1 while all other variables were measured one year later, at wave 2. BMI, body mass index; BW, body weight; MJ, megajoules

Table S2. Dietary protein intake of Māori and non-Māori LiLACS NZ participants according to frailty status at baseline (wave 2).

	**Robust**	**Pre-Frail**	**Frail**	**Total**	***p****
**Māori, n=146**					
n	38	89	19	146	
Women, n (%)	50.0 (19)	65.2 (58)	52.6 (10)	59.6 (87)	0.225
Age, (years)	83.3 (2.4)	84.0 (2.5)	84.5 (3.1)	83.9 (2.6)	0.179
Protein intake, (g/d) ¶	70.1 (27.6)	64.9 (24.9)	59.2 (29.3)	65.5 (26.2)	0.320
Men	82.46 (30.04)	75.15 (26.91)	70.40 (35.14)	76.78 (29.04)	
Women	57.69 (18.39)	59.36 (22.05)	49.20 (19.50)	57.83 (21.05)	
Protein intake, (g/kg BW/d) ¶¶	0.96 (0.43)	0.94 (0.37)	0.83 (0.45)	0.93 (0.40)	0.478
Men	1.09 (0.47)	0.98 (0.41)	0.96 (0.54)	1.01 (0.44)	
Women	0.82 (0.36)	0.93 (0.35)	0.71 (0.35)	0.88 (0.36)	
≥ 0.8 g/ kg BW/d, n (%)	52.6 (20)	61.8 (55)	52.6 (10)	58.2 (85)	0.549
Men	68.4 (13)	64.5 (20)	66.7 (6)	66.1 (39)	
Women	36.8 (7)	60.3 (35)	40.0 (4)	52.9 (46)	
≥ 1.0 g/ kg BW/d, n (%)	31.6 (12)	39.3 (35)	26.3 (5)	35.6 (52)	0.467
Men	42.1 (8)	41.9 (13)	33.3 (3)	40.7 (24)	
Women	21.1 (4)	37.9 (22)	20.0 (2)	32.2 (28)	
**Non-Māori, n=313**					
n	64	196	53	313	
Women, n (%)	43.8 (28)	52.6 (103)	54.7 (29)	51.1 (160)	0.401
Age, (years)	86.1 (0.4)	86.1 (0.4)	86.1 (0.4)	86.1 (0.4)	0.733
Protein intake, (g/d) ¶	71.7 (22.0)	68.5 (20.4)	65.9 (24.3)	68.7 (21.4)	0.332
Men	79.9 (22.6)	74.3 (22.0)	77.6 (25.8)	76.2 (22.8)	
Women	61.2 (16.1)	63.2 (17.2)	56.2 (18.4)	61.6 (17.4)	
Protein intake, (g/kg BW/d) ¶¶	1.01 (0.32)	0.98 (0.29)	0.97 (0.35)	0.99 (0.31)	0.692
Men	1.08 (0.34)	0.97 (0.29)	1.04 (0.40)	1.01 (0.32)	
Women	0.93 (0.28)	0.99 (0.28)	0.90 (0.31)	0.96 (0.29)	
≥ 0.8 g/ kg BW/d, n (%)	71.9 (46)	73.0 (143)	64.2 (34)	71.2 (223)	0.450
Men	80.6 (29)	74.2 (69)	75.0 (18)	75.8 (116)	
Women	60.7 (17)	71.8 (74)	55.2 (16)	66.9 (107)	
≥ 1.0 g/kg BW/d, n (%)	46.9 (30)	42.3 (83)	39.6 (21)	42.8 (134)	0.716
Men	55.6 (20)	38.7 (36)	50.0 (12)	44.4 (68)	
Women	35.7 (10)	45.6 (47)	31.0 (9)	41.2 (66)	

Entries are means ± SD unless mentioned.

* Unadjusted non-difference between frailty states by chi-square test (categorical variables) or one-way ANOVA (continuous variables).

¶ Estimated average requirement (EAR) and recommended dietary intakes (RDI) for adults >70 years: Men: 65g/day and 81g/day. Women: 46g/day and 57g/day [24]

¶¶ The EAR and RDI for adults >70 years: Men: 0.86g/kg BW/day and 1.07g/kg BW/day. Women: 0.75g/kg BW/day and 0.94g/kg BW/day [24]

Table S3. Wave 2 characteristics by frailty state transition and to death.

	Deteriorate				Improve	
	Robust→Prefrail	Prefrail→Frail	Prefrail→Death	Frail→Death	Frail→Prefrail	Prefrail→Robust
n	96	101	87	63	68	73
Women n (%)	56 (58.3)	60 (59.4)	39 (44.8)	32 (50.8)	42 (61.8)	33 (45.2)
Age (years)	85.6 (1.3)	85.6 (1.4)	85.5 (1.9)	85.5 (1.8)	85.8 (1.4)	85.3 (1.7)
Māori, n (%)	19 (19.8)	21 (20.8)	34 (39.1)	17 (27.0)	13 (19.1)	20 (27.4)
Education n (%)						
Primary or no schooling	12 (12.5)	14 (13.9)	27 (31.0)	19 (30.2)	6 (8.8)	11 (15.5)
Secondary school, no qualification	39 (40.6)	40 (39.6)	28 (32.2)	19 (30.2)	27 (39.7)	26 (36.6)
Secondary school, qualification	17 (17.7)	19 (18.8)	15 (17.2)	12 (19.0)	16 (23.5)	17 (23.9)
Trade, occupational	13 (13.5)	13 (12.9)	8 (9.2)	4 (6.3)	10 (14.7)	5 (7.0)
Tertiary qualification	15 (15.6)	15 (14.9)	9 (10.3)	9 (14.3)	9 (13.2)	12 (16.9)
Co-morbidity	3.3 (1.8)	3.3 (1.8)	3.2 (1.9)	3.7 (2.2)	3.8 (2.1)	2.1 (1.6)
Body weight (kg)	72.2 (12.8)	72.4 (13.6)	71.9 (13.3)	72.0 (13.6)	70.7 (14.3)	71.6 (12.3)
BMI (kg/m^2)	27.8 (4.6)	27.9 (4.8)	27.2 (4.4)	27.4 (5.4)	27.4 (4.9)	26.5 (3.4)
Energy intake (MJ/d)	7.2 (2.3)	7.1 (2.4)	7.3 (2.0)	7.0 (2.2)	7.3 (2.6)	7.6 (2.3)
Protein intake (g/d)	67.9 (24.2)	66.7 (24.4)	69.9 (23.6)	66.1 (25.1)	67.9 (27.2)	69.2 (22.4)
Protein intake (g/ kg BW/d)	1.0 (0.3)	0.9 (0.3)	1.0 (0.4)	0.9 (0.4)	1.0 (0.4)	1.0 (0.3)
≥ 0.8, n (%)	62 (64.6)	63 (62.4)	62 (71.3)	37 (58.7)	44 (64.7)	49 (67.1)
≥ 1.0, n (%)	41 (42.7)	42 (41.6)	37 (42.5)	24 (38.1)	30 (44.1)	31 (42.5)
Carbohydrate intake (g/d)	191.3 (57.5)	188.7 (58.2)	192.0 (56.0)	185.9 (56.9)	191.8 (62.2)	199.1 (58.9)
Fat intake (g/d)	70.6 (31.6)	69.1 (31.9)	72.1 (28.0)	68.9 (27.7)	73.7 (40.3)	75.6 (30.2)

These categories represent at least one transition, and some participants had the same transition twice. Further, one participant may have had more than one different transition and, therefore, observations are not independent. Ethnicity refers to Māori and non-Māori. Co-morbidity is the sum of 19 chronic diseases and was measured at wave 1 while all other variables were measured one year later, at wave 2. Entries are means ± SD unless mentioned. BMI, body mass index; BW, body weight; MJ, megajoules

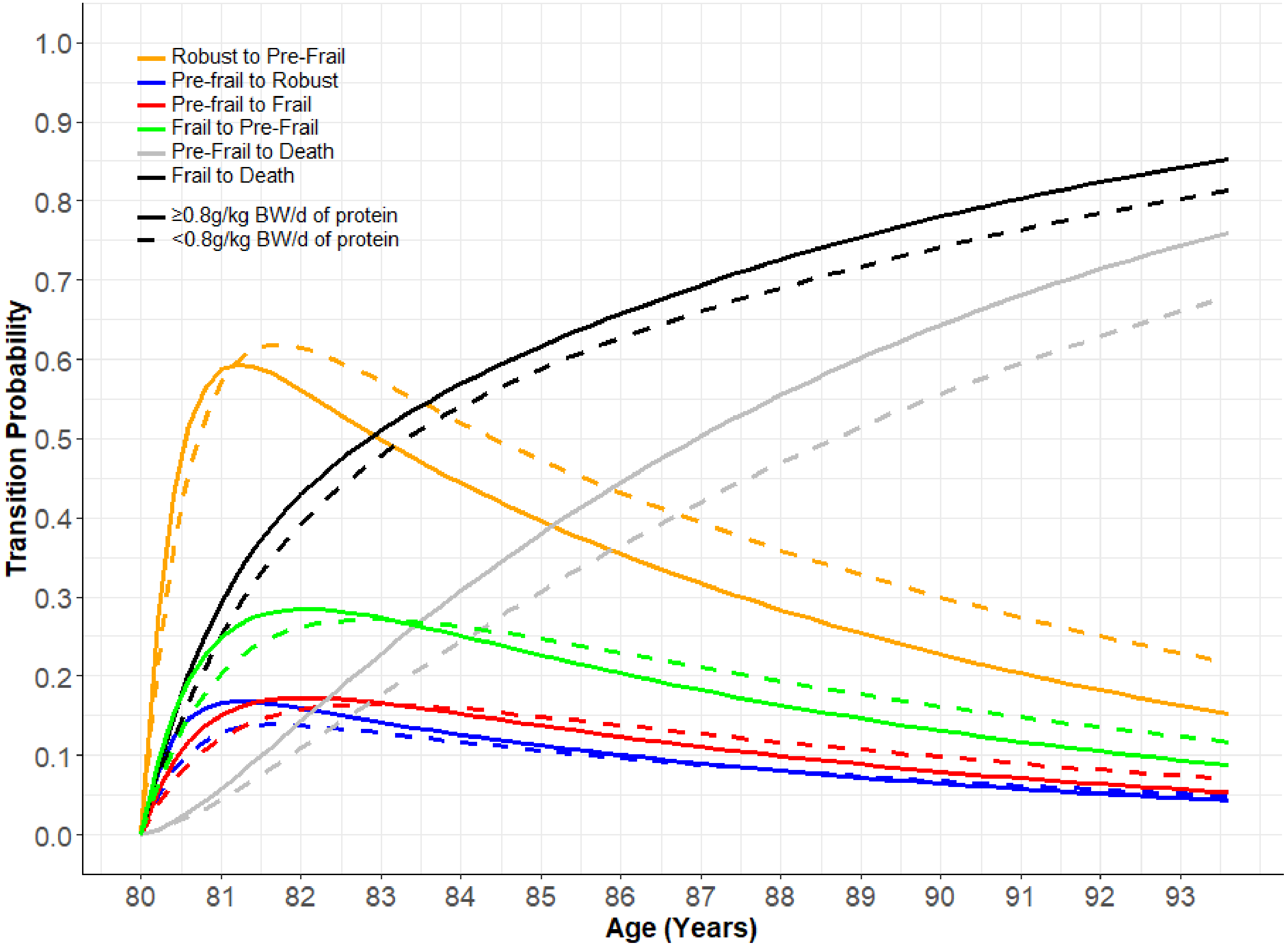

Figure S1. Transition probability between frailty states (colour coded) by < 0.8g/ kg body weight of protein intake per day (dashed line type) or ≥ 0.8g/ kg body weight of protein intake per day (full line type) by age
